# Internet‐Based Interventions in Quality of Life Assessments Among Women Living With Breast Cancer: A Systematic and Meta‐Analytic Approach

**DOI:** 10.1002/cnr2.70358

**Published:** 2025-10-31

**Authors:** Elsa Vitale, Kurvatteppa Halemani, Asha Shetty, Annarita Fanizzi, Samantha Bove, Maria Colomba Comes, Raffaella Massafra

**Affiliations:** ^1^ Scientific Directorate IRCCS Istituto Tumori “Giovanni Paolo II” Bari Italy; ^2^ College of Nursing All India Institute of Medical Sciences (AIIMS) Raebareli India; ^3^ College of Nursing All India Institute of Medical Sciences Bhubaneswar India; ^4^ Laboratorio di Bioinformatica e Biostatistica IRCCS Istituto Tumori “Giovanni Paolo II” Bari Italy

**Keywords:** assessment, breast cancer, internet‐based intervention, meta‐analysis, quality of life

## Abstract

**Introduction:**

Internet‐based interventions have become a necessity in providing care. To assess quality‐of‐life perceptions among women living with breast cancer (BC) receiving any type of internet‐based treatments.

**Methods:**

The present systematic review and meta‐analysis were registered in PROSPERO with the ID no. CRD42024498834. All internet‐based interventional studies among women living with BC assessing their quality of life perceptions before and after the intervention or between the usual and the intervention group were included. We consulted the British Nursing Database, CINHAL, Embase, Medline, Nursing & Allied Database, PubMed, Scopus, and Web of Science databases. Quality of life perceptions were assessed thanks to the Functional Assessment of Cancer Therapy–Breast (FACT‐B) and the EORTC‐QLQ‐core Questionnaire (EORTC‐QLQ‐C30).

**Results:**

The heterogeneity test findings of the eight studies exploring the FACT‐B suggested that there was a significant heterogeneity among the selected studies (*I* ^2^ = 68%, *p* = 0.002). The FACT‐B mean score among women living with BC was 0.31 (95% CI: 0.08–0.53). The heterogeneity test among the four studies exploring the EORTC‐QLQ‐C30 score revealed that there was a significant heterogeneity among the selected studies (*I* ^2^ = 74%, *p* = 0.009). The EORTC‐QLQ‐C30 mean score was 0.24 (95% CI: −0.11 to 0.58).

**Conclusion:**

Internet‐based interventions could ameliorate quality of life perceptions since most of the enrolled participants revealed as complicated the home community management allowing regular participation.

## 1 Introduction

Breast cancer (BC) has been recognized as the most frequent cancer disease among females, by encountering nearly 2.3 million women suffering worldwide and 685 000 deaths, and it is estimated that by the end of 2020, nearly 7.8 million female survivors had a BC diagnosis in their past 5 years, too [1]. Thus, it should be considered BC as a very interesting subject in our present society. BC usually impacts fatigue patients' perceptions and functioning and thus, their health‐related quality perceptions, recognizing the most usual recorded late effect in all BC available treatments [2].

In this scenario, most mobile technologies are increasing to ameliorate BC patients' self‐management in several aspects of women's lives, such as fatigue, quality‐of‐life perceptions, and women's functioning.

Compared with usual care, internet‐based interventions seemed to be more suitable without any limitations by time and space, reaching more patients [3]. Past studies have reported advantages in BC patients in self‐management attitudes [4], functional compliance [5], self‐efficacy [6], anxiety and depression [7], social support [4], and quality of life (QoL) perception [8]. However, the findings of previous studies were controversial [9, 10].

Thus, internet‐based interventions become a necessity in providing care. Literature included internet‐based interventions in the “digital health” topic by embracing all the health facilities and information that help to handle disease and its related risks [11, 12]. Health facilities have been developed thanks to several internet‐related approaches, such as mobile health, telemedicine, telemonitoring, digital therapeutics, and related health analytics for health [13], mainly via websites [14, 15], mobile applications (apps) [16, 17], and social media software, like WeChat [18, 19] and Facebook [20]. In addition, the Covid‐19 pandemic has advanced the progress and improvement in digital health applications to supply the most efficient care [21]. In this aspect, Basch et al. [22] integrated an internet‐based patient‐reported outcome checking in symptoms recording during cancer treatment and showed lower levels in their quality‐of‐life perceptions, with improvements in readmission and compliance in treatment received. Evidence highlighted several internet‐based interventions in BC [17], prostate cancer [16], melanoma [18], and hematologic malignancy [23] diseases to cope with physical and emotional symptoms, health attitudes, self‐efficacy [15], or oral anticancer therapy adherence [24].

Considering what has been reported here, the present systematic and meta‐analyses aimed to assess quality‐of‐life perceptions among women living with BC who received any typology of internet‐based treatments.

## 2 Materials and Methods

### 2.1 Data Sources

To perform the present systematic review and meta‐analysis, we consulted online British Nursing Database, CINAHL, Embase, Medline, Nursing & Allied Database, PubMed, Scopus, and Web of Science databases (File S1).

### 2.2 Search Strategy

This systematic review and meta‐analysis were performed according to the Preferred Reporting Items for Systematic Reviews and Meta‐Analyses (PRISMA) checklist [25]. The review protocol was registered in PROSPERO with the ID no. CRD42024498834.

Search strategy was carried out using a combination of keywords and MeSH terms, and a research question was created thanks to the “Population‐Intervention‐Outcome” (PIO) approach (Table 1) [25].

**TABLE 1:** The PIO tool for the scoping review.

Population	Breast cancer women
Intervention	Internet‐based intervention
Outcome	Functional and Quality of Life assessments

### 2.3 Eligibility Criteria

We considered only original trials published in the English language, focusing on QoL assessments in women living with BC who received any typology of internet‐based interventions.

Trials that have a qualitative assessment of QoL perceptions without a quantitative tool were excluded from this systematic review.

All duplicated studies and non‐comparison studies or studies involving men living with BC, were excluded from additional analysis.

### 2.4 Data Extraction Process

**FIGURE 1:**
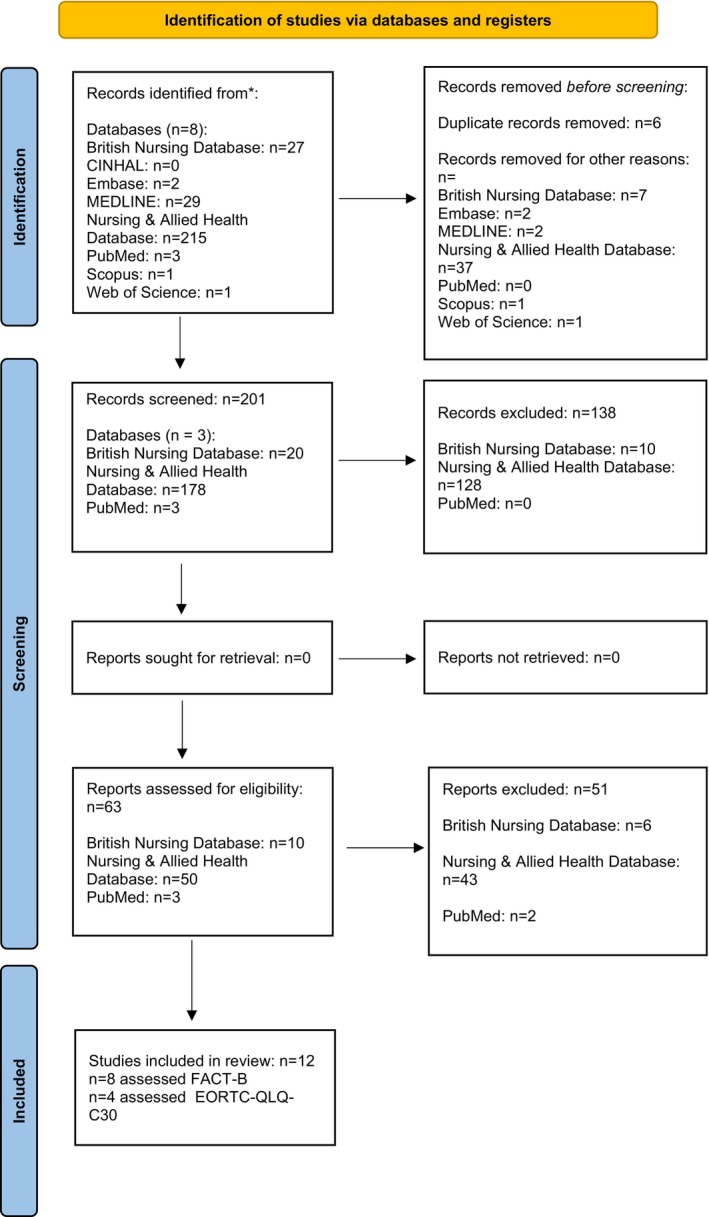
The PRISMA flow diagram.

Initially, titles and abstracts were identified and uploaded to remove duplicates, and a total of 201 records were screened. Then, two independent reviewers (E.V. and K.H.) screened titles and abstracts, and all the full texts were assessed for eligibility, and 138 studies were removed. Thus, a total of 63 eligible records were found. In addition, reviewers included in the present literature research were further hand‐searched and assessed to not exclude any potentially valuable study. However, 51 out of these 63 trials were excluded as they did not meet the inclusion review criteria. Finally, the remaining 12 trials were considered for the present systematic review and meta‐analysis. A summary of the article screening process was displayed in the PRISMA flow diagram (Figure 1).

All the included studies were illustrated considering study characteristics (author, year of publication, aim, design, setting, and experimental intervention), participants (age, BC stage, type of treatments performed), and QoL perceptions both according to the FACT‐B and EORTC‐QLQ‐C30 scores expressed as mean and standard deviations [26, 27].

### 2.5 Outcomes

The present systematic review and meta‐analysis included all internet‐based interventional studies among women living with BC assessing their QoL perceptions before and after the intervention or between the usual and the intervention group. Evidence collected allowed us to assess QoL perceptions thanks to the Functional Assessment of Cancer Therapy–Breast (FACT‐B) and the EORTC‐QLQ‐core Questionnaire (EORTC‐QLQ‐C30) [26, 27]. The FACT‐B included a total of 37 items assessing five dimensions of the Health‐Related Quality of Life (HRQOL) among women living with BC, such as physical, social, emotional, and functional. Significant sensitivity was shown from a larger sample with internal consistency FACT‐B total score_*α* = 0.90, with also good test–retest reliability, convergent, divergent, and group validity, too [26, 27].

The EORTC Core Quality of Life questionnaire (EORTC‐QLQ‐C30) covered a harmonized system to assess the health QoL in cancer patients involved in international clinical trials by embracing five dimensions, namely: physical, role, cognitive, emotional, and social; plus three symptom assessments, namely: fatigue, pain, nausea and vomiting; and a global health perception with further symptoms usually added in cancer patients, such as: dyspnea, loss of appetite, insomnia, constipation and diarrhea, and perceived financial consequences in the disease experience [26]. The EORTC‐QLQ‐C30 questionnaire showed good internal consistency with *α* ≥ 0.70 [26].

### 2.6 Quality Assessment and Risk of Bias

**FIGURE 2:**
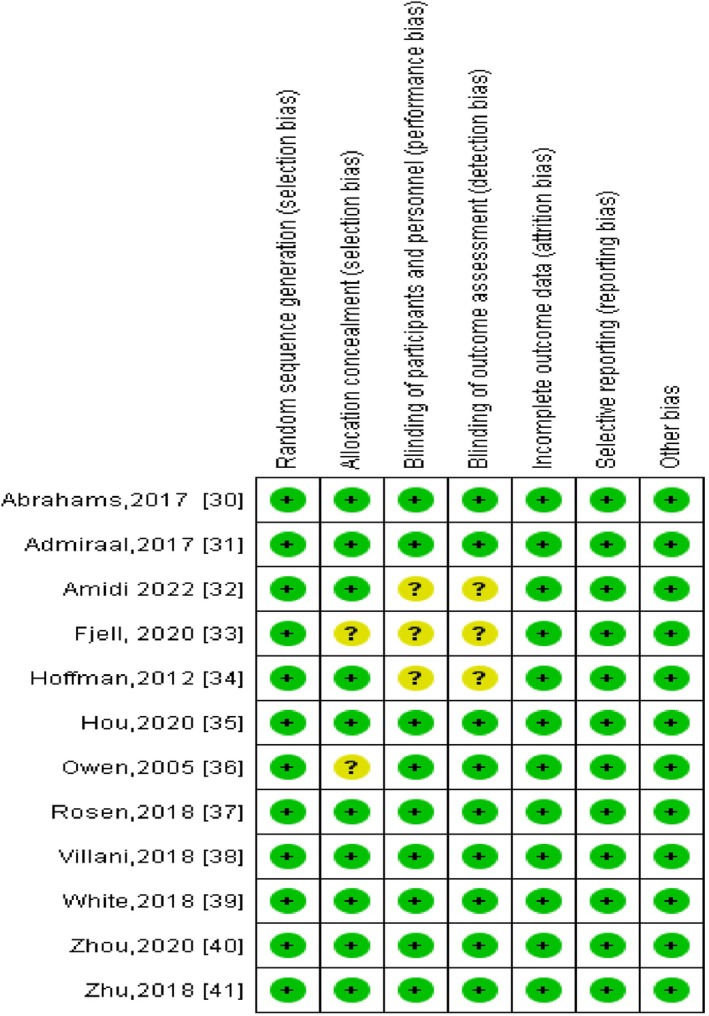
Risk of bias summary of the included studies.

All the studies included in the present systematic review were assessed in their methodological qualities thanks to the RoB tool [28], as shown in Figure 2. The RoB 2 tool included a total of five specific fields, namely: randomization process, deviations from the intended interventions, missing outcome data, measurement of the outcome, and selection of the reported results. A score of low, medium, or high risk of bias was assessed to classify our meta‐analysis for each dimension assessed.

### 2.7 Data Synthesis

The primary outcomes of the included studies (functional and QoL) were compared between participants who received Internet‐based interventions and the participants in the control group who received routine/usual care. The results of the continuous variables (mean and standard deviation) were pooled using a random effects model. The effect size was calculated using standardized mean difference (SMD) with a 95% confidence interval (CI) for each individual study. The heterogeneity of the included trials was evaluated using the *I* ^2^ value, categorizing it as low (< 50%), moderate (50%–75%), or high (> 75%) heterogeneity. The data were analyzed and pooled using RevMan v5.3 software.

## 3 Results

From our literature research, studies were assessed according to the FACT‐B and the EORTC‐QLQ‐C30 scores. A total of 12 studies were summarized [7, 29, 30, 31, 32, 33, 34, 35, 36, 37, 38, 39] according to their internet‐based interventions by considering study characteristics (author, year of publication, aim, design, setting, and experimental intervention), participants (age, BC stage, type of treatments performed), and outcomes both in FACT‐B (Table 2) and EORTC‐QLQ‐C30 scores (Table 3).

**TABLE 2:** Study characteristics of all the selected articles exploring the FACT‐B assessment (*n* = 8).

Authors name (year), country	Research design	Sample characteristics			Interventions	Assessment scales	Study outcomes
		Sample size	Inclusion criteria	Exclusion criteria			
Amidi et al. (2022), Denmark [29]	RCT	CG: 54 IG: 77 T: 131	BC women Age ≥ 18 years Hospitalization regimen	Recurrence BC Physical comorbidity Sleep disorders Not Danish language native	CG: Usual care IG: Online program of 6 modules: treatment rationale, stimulus control, cognitive restructuring, sleep hygiene, and relapse prevention	BDI‐II FACIT‐F ISI PSQ	IG reported improvements in sleep quality
Hoffmann et al. (2012), UK [38]	RCT	CG: 115 IG: 114 T: 229	BC women Age: 18–80 years Surgery, chemotherapy, or radiotherapy treatment	Not English language native Inability to give consent	CG: Usual care IG: The MBSR yoga‐based stretches, meditation, group discussions, and didactic teaching program	FACT‐B FACT‐ES POMS	IG showed significant on breast‐ and endocrine‐related fatigue
Owen et al. (2005), USA [39]	RCT	CG: 30 IG: 32 T: 62	BC women	Multiple illness	CG: Usual care IG: Self‐guided, Internet‐based coping‐skills training	FACT‐B, IES MSAS Physical well‐being	GI significantly improved psychological well‐being
Rosen et al. (2018), USA [32]	RCT	CG: 55 IG: 57 T: 112	BC women Age ≥ 25 years Ability to use a smartphone or a tablet	NR	CG: Usual care IG: A 6‐month AMT subscription to learn and practice mindfulness skills for 10 days	BRIEF FACT‐B MAAS QOL	AMT enhanced QOL
Villani et al. (2018), Italy [33]	RCT	CG: 14 IG: 15 T: 29	BC women Age ≥ 55 years Radical surgery Negative staging with metastases	NR	CG: Usual care IG: E‐intervention live‐video; interviews related to cancer experiences, chemotherapy, side effects, modeling to cope with physical and emotional changes	ERQ FACT‐B	eHealth interventions gave a challenge and an opportunity to learn
White et al. (2018), Australia [34]	RCT	CG: 202 IG: 177 T: 379	BC women Age: 18–50 years BC stage: I or II English speaking	Women with a previous diagnosis of cancer or a prognosis less than 18 months	CG: Usual care IG: Self‐directed website divided into pages covering emotional responses, support services, family responses, and life after cancer	FACT‐B Supportive Care Need Survey‐Breast Cancer	GI recognized a high number of unmet needs
Zhou et al. (2020), China [7]	RCT	CG: 55 IG: 56 T: 111	BC women Age ≥ 18 years BC stage: I–III	Comorbidity of several illnesses	CG: Usual care IG: WeChat‐based multimodal nursing program administered during hospitalization and 6 months post‐surgery	FACT‐B NRS at pre‐ and post‐surgery	WeChat‐based multimodal nursing program significantly improved QOL in postoperative course
Zhu et al. (2018), Australia [35]	RCT	CG: 57 IG: 57 T: 114	BC women Ability to access the internet through the mobile phone Mandarin speaking	Women with concurrent major physical or mental Mental diseases	CG: Usual care IG: The BCS program, including: Learning forum, Discussion forum, ask‐the‐Expert forum, and Personal Stories forum	SICPA–Chinese version MSPSS–Chinese version	IG recorded better self‐efficacy, symptom interference, and QOL during chemotherapy

Abbreviations: AMT, App‐delivered mindfulness training; BC, breast cancer; BRIEF, The Brief Health Literacy Screening Tool; CG, no. of participants assigned to the control group; eHEALS, eHealth Literacy Scale; EORTC, European Organization for Research and Treatment of Cancer; ERQ, Emotion Regulation Questionnaire; FACT‐B, Functional Assessment of Cancer Treatment‐B; FACT‐ES, Functional Assessment of Cancer Therapy‐Endocrine Symptoms; FACIT–Fatigue, Functional Assessment of Chronic Illness Therapy—Fatigue Scale; HADS, Hospital Anxiety and Depression Scale; IG, no. of participants assigned to the interventional group; IES, Impact of Events scale; ISI, insomnia severity index; MAAS, Mindful Attention Awareness Scale; MBSR, mindfulness‐based stress reduction; MSAS, Memorial Symptom Assessment Scale; NR, not reported; NRS, Numerical Rating Scale; POMS, Profile of Mood States Questionnaire; PSQ, Pittsburg Sleep Quality Index; QLQ‐BR, Breast Cancer‐Specific Quality‐of‐Life Questionnaire; QLQ‐C, Quality‐of‐Life Questionnaire Core; QOL, Quality‐of‐Life; SICPA, Stanford Inventory of Cancer Patient Adjustment; SIT, stress inoculation training; T, no. total participants; VCR, Victorian Cancer Registry.

**TABLE 3:** Study characteristics of all the selected articles exploring the EORTC‐QLQ‐C30 assessment (*n* = 4).

Authors name (year), Country	Research design	Sample characteristics			Interventions	Assessment scales	Study outcomes
		Sample size	Inclusion criteria	Exclusion criteria			
Abrahams et al. (2017), Netherland [37]	RCT	CG: 66 IG: 66 T: 132	BC women Age: 18–65 years Dutch speaking Ability to use a computer and the Internet	BC women with physical and psychological comorbidity	CG: Usual care IG: Cognitive behavioral treatment model of precipitating and perpetuating factors of fatigue and malignancy	EORTC Fatigue: Sickness scale	Cognitive behavioral treatment is effective in reduced severe fatigue and related symptoms
Admiraal et al. (2017), Netherlands [36]	RCT	CG: 69 IG: 69 T: 138	BC women over the past 8 years Dutch speaking Ability to assess email	BC women with recurrent and/or metastasis	CG: Usual care IG: ENCOURAGE program with fully automated information problem‐solving strategies for 6 and 12 weeks	EORTC FACT‐B‐Chinese version MDASI HADS	The ENCOURAGE could be a promising program for BC women
Fjell et al. (2020), Sweden [30]	RCT	CG: 75 IG: 74 T: 149	BC women Age ≥ 18 years Swedish speaking	BC women with physical and psychological comorbidity	CG: Usual care IG: The Interaktor application, including self‐reporting of 14 common symptom‐management during chemotherapy	QOL Self‐reported symptoms	GI reported reduced symptom burden and improved emotional functioning
Hou et al. (2020), China [31]	RCT	CG: 59 IG: 53 T: 112	BC women Age: 20–65 years BC stage: 0–III Ability to use a mobile phone Chinese speaking	NR	CG: Usual care IG: BCSMS app included psychosocial support	EORTC QLQ‐BR23	The BCSMS app enhanced self‐management and QOL

Abbreviations: AMT, App‐delivered mindfulness training; BC, breast cancer; BDI‐II, Beck Depression Inventory; BRIEF, The Brief Health Literacy Screening Tool; CG, no. of participants assigned to the control group; eHEALS, eHealth Literacy Scale; EORTC, European Organization for Research and Treatment of Cancer; ERQ, Emotion Regulation Questionnaire; FACT‐B, Functional Assessment of Cancer Therapy‐Breast; FACT‐ES, Functional Assessment of Cancer Therapy‐Endocrine Symptoms; HADS, Hospital Anxiety and Depression Scale; IES, Impact of Events Scale; IG, no. of participants assigned to the interventional group; ISI, Insomnia Severity Index; MBSR, mindfulness‐based stress reduction; MAAS, Mindful Attention Awareness Scale; MDASI, M.D. Anderson Symptom Inventory; MSAS, Memorial Symptom Assessment Scale; PSQ, Pittsburg Sleep Quality Index; QLQ‐BR, Breast Cancer‐Specific Quality‐of‐Life Questionnaire; QLQ‐C, Quality‐of‐Life Questionnaire Core; QOL, Quality of life; VCR, Victorian Cancer Registry; SICPA, Stanford Inventory of Cancer Patient Adjustment; SIT, stress inoculation training; T, no. total participants.

### 3.1 Studies Exploring the FACT‐B Assessment

Eight studies were screened, which assessed the FACT‐B score after an internet‐based intervention [7, 29, 32, 33, 34, 35, 38, 39] (Table 2).

The internet‐delivered cognitive behavioral therapy for insomnia (e‐CBT‐I) was introduced among women living with BC by reporting satisfactory results at 12 months posttreatment, with the recommendation of the antihormonal therapy for at least 10 years and improvements in cancer‐related fatigue (95% CI: 0.30–0.66) [30]. In this regard, CBT‐I has just been recognized as a versatile treatment methodology covering a mixture of different approaches, like stimulus control therapy, sleep restriction therapy, relaxation therapy (RT), cognitive therapy (CT), and sleep hygiene education (SHE) to reduce the time in bed when participants are not sleeping and to improve healthy sleep enduring over time [40].

Another therapeutic approach delivered in online mode was the mindfulness‐based stress reduction (MBSR) approach, which seemed to ameliorate mood, breast‐ and endocrine‐specific QoL, and well‐being [39]. Encouraging results were shown in a randomized‐controlled trial recruiting 229 women living with BC after surgery, chemotherapy, and radiotherapy. Patients were assessed at Weeks 0, 8, and 12. MBSR seemed to ameliorate long‐term emotional and physical adverse effects during medical treatments, including endocrine treatments that were also prolonged at 3 months [39]. Moreover, mindfulness training was improved thanks to a mobile app‐delivered strategy. Women using this mobile app recorded greater and significant QOL levels [34]. A self‐guided, internet‐based coping skills training approach was improved among women living with BC in their QoL perceptions [33], considering that internet‐based approaches seemed to be effective in providing psychological support for cancer survivors. This approach reported a significant improvement in self‐reported health condition and treatment in perceived health, with an important reduction in sadness and anxiety, cognitive processing, and health‐related demonstrations [33]. Another internet‐based and self‐guided approach adopted for women living with BC was a 2‐week e‐health stress inoculation training (SIT) intervention on emotion regulation and cancer‐related well‐being. Thanks to SIT, patients recorded significant reductions in emotional inhibition and increased cancer‐related psychological well‐being in leisure, along with a reduction of anxiety and a satisfactory level of adoption in their usual psychosocial care path within a multi‐stratified care model [7]. A WeChat‐based multimodal nursing program was implemented for women living with BC reporting pain, fatigue, and sleep disturbance. The WeChat‐based multimodal nursing program included 6 months of usual nursing care plus WeChat intervention. Data reported a significant improvement in the FACT‐B, as well as in social, family, and functional well‐being (*p* < 0.05) in clinical and transitional nursing care [7]. Another web‐based program was scheduled to support women in discovering the information and facilities that were considered necessary. A total of 202 participants belonged to the Intervention Group and entered the website at least once for a mean of 19 min. Total QoL was better in the intervention group than in the control one. However, most of the unmet needs were recognized, as further approaches assessing these needs were necessary [38]. In this regard, Zhou et al. [7] recognized that women living with BC who experienced chemotherapy reported unmet supportive care needs. Therefore, an app‐based BC e‐support program was created to guide women living with BC in their self‐efficacy, social support, symptom distress, QoL, anxiety, and depression. This e‐support program guided women for 12 weeks in 4 cycles of chemotherapy, with a significant amelioration in QoL (95% CI: 0.77–12.50; *p* = 0.03) by showing successful and undoubtedly increased involvement in improving women's self‐effectiveness, symptom intrusion, and QoL during chemotherapy [7].

### 3.2 Studies Exploring the EORTC‐QLQ‐C30 Assessment

Four studies assessed BC women's QoL thanks to the EORTC‐QLQ‐C30 score after experiencing an internet‐based intervention [30, 31, 36, 37] (Table 3). An internet‐based cognitive behavioral therapy (I‐CBT) was improved among BC patients, and their QoL was assessed. The I‐CBT program included a total of three face‐to‐face sessions and eight other web‐based modules, followed by further electronic consultations, like emails, telephone, or video consultations. The I‐CBT seemed to have positive effects in severely fatigued survivors of BC [36]. Another web‐based tailored psychoeducational intervention called “ENCOURAGE” was performed by Admiraal et al. [36] to support women living with BC in their unmet requirements in information and psychosocial needs after treatment completion. The ENCOURAGE program provided a total of 12 weeks of access to improve problem‐solving approaches, resources, and services, and reported greater optimism and control attitudes in women living with BC involved. A breast cancer self‐management support (BCSMS) through the mHealth app was designed for women living with BC, and the QoL was assessed and recorded at significantly higher levels among the experimental group versus the control group at 3 months [32]. However, there was a lack of apps for women living with BC for immediate clinical management, as explained by Hou et al. [31], especially among patients at home. As the BCSMS app, the Interaktor app could also represent a disease self‐management tool that regularly monitors patients and improves health‐related QoL [31], also in symptom management, like nausea, vomiting, feeling sad, appetite loss, and constipation.

### 3.3 Meta‐Analysis Results

**FIGURE 3:**
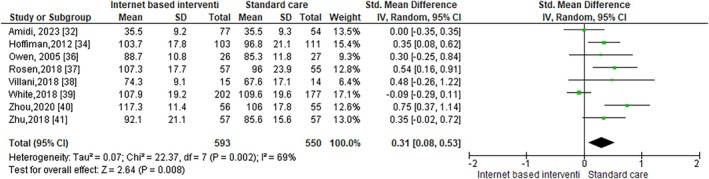
Forest Plot for the FACT‐B assessment among women living with BC.

The heterogeneity test findings of the eight studies exploring the FACT‐B among women living with BC [7, 29, 32, 33, 34, 35, 38, 39] suggested that there was significant heterogeneity among the studies selected (*I* ^2^ = 68%, *p* = 0.002). The random effect model assessed for the FACT‐B mean score was 0.31 (95% CI: 0.08–0.53) (Figure 3).

**FIGURE 4:**
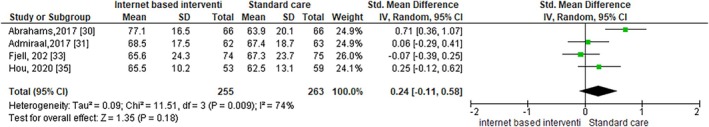
Forest plot for the EORTC‐QLQ‐C30 assessment among women living with BC.

The heterogeneity test findings of the four studies exploring the EORTC‐QLQ‐C30 score [29, 31, 32, 36] among women living with BC revealed that there was a significant heterogeneity among the studies selected (*I* ^2^ = 74%, *p* = 0.009). The random effects model assessed for the EORTC‐QLQ‐C30 mean score was 0.24 (95% CI: −0.11 to 0.58) (Figure 4).

## 4 Discussion

A total of 12 studies were summarized [7, 29, 30, 31, 32, 33, 34, 35, 36, 37, 38, 39] based on their internet‐based interventions, considering the FACT‐B and the EORTC‐QLQ‐C30 scores. Data collected highlighted a significant trend in both preference and outcome for in‐person or no online interventions [7, 29, 30, 31, 32, 33, 34, 35, 36, 39]. However, we should consider that cancer disease frequently induces fatigue, and no standard treatments were scheduled [41] with the two co‐occurring symptoms in its related etiology [42]. Live‐video interviews were evaluated as helpful and satisfying, also in brief mindfulness meditation programs, which could be favorably combined in self‐support interventions through new technologies and mobile apps [43, 44]. Although internet‐based interventions may improve fatigue outcomes [42], most participants showed retention (67% completed all measures) [45], since several participants revealed that the home community management [30] was complicated over the difficulty of allowing regular participation, like the consent process and lengthy questionnaires to complete. Generally, retraction seemed to be greater among participants recording important baseline QOL and better pain intrusion [34]. In addition, early BC experience showed that age could be judged as an influencing factor in patients' distress [46], which in turn could be recognized as one of the main factors of vulnerability to long‐term distress [47]. Positive effects in internet‐based programs could entail participation for several weeks and represent a probable cause in the study's shortcoming of participation in the intervention group, who reported lower levels of engagement with the internet‐based facilities. Thus, the access to all the internet‐based interventions proposed in all the studies included necessary access to the information section, which needed time to read and then to speedily use [35]. By considering that women living with BC were doubtful to cope with possible adverse effects in the posttreatment phase, like severe nausea, vomiting, hair loss, and fatigue, a pretreatment counseling which could help women to improve their nursing knowledge and advance engaged approaches to better deal with the prescribed treatment. This approach could ameliorate the online participation for these patients [48], with promising outcomes in emotion regulation and cancer‐related well‐being appeared winning [49]. Therefore, patients' engagement appeared essential in internet‐based interventions [50]. For example, in the Amidi et al. study [29], sleep outcomes were considered as predictors of decreased fatigue levels and reported a significant amelioration in fatigue and sleep disorders, too [51]. These results seemed to be equally promising as the face‐to‐face approach [52] at 12 months after intervention. Even interesting findings were found in Hofmann et al.'s study [38] regarding the MBSR intervention in mood, anxiety, depression, anger, vigor, fatigue, and confusion, QoL, emotional, physical, social, functional, and general well‐being, which agreed with the current online interventional outcomes [53]. However, the recommendation for women living with BC to adopt MBSR to improve mood and QoL could also involve endocrine symptoms [39], too. In this regard, there was a tendency to imagine an emotional well‐being improvement and related QoL in internet‐based interventions [33]. Conversely, Owen et al. [39] showed no significant improvements in women living with BC with three general patterns adopted, like: deeply ventilation of feelings linked to anxiety and sadness, and important achievements to recognize all the cancer‐related treatment pathway and its concerns [54]. Stanton et al. [55] explained a theoretical relationship between emotional strategic coping to stressful conditions, which might improve the reassessment of the stressor, empower social relationships, and improve knowledge of individual actionable outcomes [33]. The e‐health involvement empowered at least three adaptive approaches usually linked to psychological well‐being, like acceptance, emotional elaboration, and expression, in order to facilitate patients' communication [56], accomplished by relaxation and meditation to reduce patients' stress [57, 58]. This approach was observed in the AMT program through free access to mindfulness app content [7]. The WeChat‐based multimodal nursing was employed during the postoperative follow‐up time in women living with BC and reported significantly lower effects in women living with BC [7]. On the other hand, the BCS usage found no long‐term effects for women living with BC at 6 months [55]. The app‐based BCS program could supply qualified functions and perform similar effects as computer‐based programs [59], whereas women enrolled registered different goals at 6 months, since most of them had concluded chemotherapy and returned to their habitual physical, psychological, and social lives at 6 months. Maybe the BCS program could focus on chemotherapy support without any sufficient records for the posttreatment phase, which should be considered for future app‐based studies.

As regards the quality‐of‐life assessment, in the Abrahams et al. study [37], ICBT therapists helped patients through the website and by email by suggesting mandatory and optional modules to read. The web‐based intervention, compared to the face‐to‐face CBT for severe fatigue, registered similar effects in decreasing severe fatigue [60]. This result was also obtained by a previous meta‐analysis showing the effects of face‐to‐face CBT compared to therapist‐guided ICBT, and findings suggested no significant differences in patients suffering from psychiatric and somatic disorders [61]. On the other hand, Yun et al. [62] reported an important amelioration in fatigue goals by using a web‐based self‐management training program. Also, in the ENCOURAGE program [29], no positive effects compared with the standard care were observed, especially among women living with BC with optimism and control for the future. Thus, the program could report positive effects only for patients' subgroups [29], maybe directly related to treatment or emotional symptoms [63, 64]. In addition, Kondylakis et al. [65] showed emotional improvement in utilizing a digital self‐management application. However, the emotional functioning assessed by the EORTC QLQ‐C30 could be influenced during the treatment [66] or maybe an effect of the remaining symptom burden [67]. Also, the BCSMS app users recorded significantly higher scores in their functional assessments [32]. Based on these results, mobile health apps could successfully encourage patients in their chronic disease self‐handling [68]. Other evidence has shown that mobile health apps were linked to knowledge amelioration of the disease and self‐handling, covering self‐efficacy with performing functional exercises during and after chemotherapy [1], thanks to a personalized approach [32].

## 5 Conclusion

From our systematic review and meta‐analysis, selected studies underlined a significant trend in both preference and outcome for in‐person interventions. These results seemed to be equally promising as the face‐to‐face approach, both in mood, anxiety, depression, anger, vigor, fatigue, and confusion, QoL, emotional, physical, social, functional, and general wellbeing. Internet‐based interventions could ameliorate QoL perceptions, since most of the enrolled participants revealed the home community management as complicated, allowing regular participation. However, it should be considered that the total number of studies (*n* = 12) was not good enough to generalize our conceptual results (eight for FACT and four for EORTC scales), so further studies will be needed to better understand our findings.

## Author Contributions

**Elsa Vitale:** conceptualization (lead), data curation (lead), investigation (lead), methodology (lead), resources (equal), supervision (lead), visualization (lead), writing – original draft (lead), writing – review and editing (lead). **Kurvatteppa Halemani:** conceptualization (supporting), investigation (equal), methodology (equal), supervision (supporting), visualization (supporting). **Asha Shetty:** data curation (equal), resources (supporting). **Annarita Fanizzi:** methodology (equal). **Samantha Bove:** investigation (equal). **Maria Colomba Comes:** data curation (equal). **Raffaella Massafra:** resources (lead).

## Disclosure

The authors affiliated with the IRCCS Istituto Tumori “Giovanni Paolo II,” Bari, are responsible for the views expressed in this article, which do not necessarily represent the Institute.

## Ethics Statement

The authors have nothing to report.

## Consent

The authors have nothing to report.

## Conflicts of Interest

The authors declare no conflicts of interest.

## Supporting information


**Data S1:** Supporting Information.

## Data Availability

Data are collected all in the File S1 and in Tables 1, 2, 3.
