# Supplementary material for: Internet‐Based Interventions in Quality of Life Assessments Among Women Living With Breast Cancer: A Systematic and Meta‐Analytic Approach
Source: Cancer Rep (Hoboken). 2025 Oct 31;8(11):e70358. doi: 10.1002/cnr2.70358 (PMC12576468; doi:10.1002/cnr2.70358)
Supplement: Supplementary file 1 — Data S1: Supporting Information. [file CNR2-8-e70358-s001.docx]

**Supplementary file 1. Search strings carried out to perform this systematic and meta-analysis study.**

| **Database** | **Results** |
| --- | --- |
| **British Nursing Database**  (Internet-Based Intervention) AND Fatigue AND Stress AND (Quality of Life) AND (breast neoplasms) | 15 |
| **CINHAL**  (Internet-Based Intervention) AND Fatigue AND Stress AND (Quality of Life) AND (breast neoplasms) | 0 |
| **EMBASE**  ('internet-based intervention'/exp OR 'internet-based intervention') AND ('fatigue'/exp OR 'fatigue') AND ('stress'/exp OR 'stress') AND ('quality of life'/exp OR 'quality of life') AND ('breast neoplasms'/exp OR 'breast neoplasms') | 2 |
| **MEDLINE**  (Internet-Based Intervention) AND Fatigue AND Stress AND (Quality of Life) AND (breast neoplasms) | 29 |
| **Nursing & Allied Health Database**  (Internet-Based Intervention) AND Fatigue AND Stress AND (Quality of Life) AND (breast neoplasms) | 8 |
| **PubMed**  Search: **((((Internet-Based Intervention) AND (Fatigue)) AND (Stress)) AND (Quality of Life)) AND (breast neoplasms)**  ("internet based intervention"[MeSH Terms] OR ("internet based"[All Fields] AND "intervention"[All Fields]) OR "internet based intervention"[All Fields] OR ("internet"[All Fields] AND "based"[All Fields] AND "intervention"[All Fields]) OR "internet based intervention"[All Fields]) AND ("fatiguability"[All Fields] OR "fatiguable"[All Fields] OR "fatigue"[MeSH Terms] OR "fatigue"[All Fields] OR "fatigued"[All Fields] OR "fatigues"[All Fields] OR "fatiguing"[All Fields] OR "fatigueability"[All Fields]) AND ("stress"[All Fields] OR "stressed"[All Fields] OR "stresses"[All Fields] OR "stressful"[All Fields] OR "stressfulness"[All Fields] OR "stressing"[All Fields]) AND ("quality of life"[MeSH Terms] OR ("quality"[All Fields] AND "life"[All Fields]) OR "quality of life"[All Fields]) AND ("breast neoplasms"[MeSH Terms] OR ("breast"[All Fields] AND "neoplasms"[All Fields]) OR "breast neoplasms"[All Fields])  **Translations**  **Internet-Based Intervention:** "internet-based intervention"[MeSH Terms] OR ("internet-based"[All Fields] AND "intervention"[All Fields]) OR "internet-based intervention"[All Fields] OR ("internet"[All Fields] AND "based"[All Fields] AND "intervention"[All Fields]) OR "internet based intervention"[All Fields]  **Fatigue:** "fatiguability"[All Fields] OR "fatiguable"[All Fields] OR "fatigue"[MeSH Terms] OR "fatigue"[All Fields] OR "fatigued"[All Fields] OR "fatigues"[All Fields] OR "fatiguing"[All Fields] OR "fatigueability"[All Fields]  **Stress:** "stress"[All Fields] OR "stressed"[All Fields] OR "stresses"[All Fields] OR "stressful"[All Fields] OR "stressfulness"[All Fields] OR "stressing"[All Fields]  **Quality of Life:** "quality of life"[MeSH Terms] OR ("quality"[All Fields] AND "life"[All Fields]) OR "quality of life"[All Fields]  **breast neoplasms:** "breast neoplasms"[MeSH Terms] OR ("breast"[All Fields] AND "neoplasms"[All Fields]) OR "breast neoplasms"[All Fields] | 3 |
| **Scopus**  ( TITLE-ABS-KEY ( internet-based AND intervention ) AND TITLE-ABS-KEY ( fatigue ) AND TITLE-ABS-KEY ( stress ) AND TITLE-ABS-KEY ( quality AND of AND life ) AND TITLE-ABS-KEY ( breast AND neoplasms ) ) | 1 |
| **WOS**  telenursing (All Fields) and breast cancer (All Fields) and randomized controlled trial (All Fields) | 1 |
